# Differentiation of Human Umbilical Cord Mesenchymal Stem Cells into Prostate-Like Epithelial Cells *In Vivo*

**DOI:** 10.1371/journal.pone.0102657

**Published:** 2014-07-23

**Authors:** Wang Li, Bo Ye, Xiao-Yan Cai, Jian-Hua Lin, Wei-Qiang Gao

**Affiliations:** 1 State Key Laboratory of Oncogenes and Related Genes, Renji-Med X Clinical Stem Cell Research Center, Ren Ji Hospital, School of Medicine, Shanghai Jiao Tong University, Shanghai, China; 2 Department of Thoracic Surgery, Shanghai Chest Hospital, Shanghai Jiao Tong University, Shanghai, China; 3 Department of Obstetrics and Gynecology, Ren Ji Hospital, School of Medicine, Shanghai Jiao Tong University, Shanghai, China; 4 School of Biomedical Engineering & Med-X Research Institute, Shanghai Jiao Tong University, Shanghai, China; Rutgers - New Jersey Medical School, United States of America

## Abstract

Although human umbilical cord mesenchymal stem cells (hUC-MSCs) have been identified as a new source of MSCs for potential application in regenerative medicine, their full potential of differentiation has not been determined. In particular, whether they have the capability to differentiate into epithelial cells of endodermal origin such as the prostate epithelial cells is unknown. Here we report that when hUC-MSCs were combined with rat urogenital sinus stromal cells (rUGSSs) and transplanted into the renal capsule in vivo, they could differentiate into prostate epithelial-like cells that could be verified by prostate epithelial cell-specific markers including the prostate specific antigen. The prostatic glandular structures formed in vivo displayed similar cellular architecture with lumens and branching features as seen for a normal prostate. In addition, the human origin of the hUC-MSCs was confirmed by immunocytochemistry for human nuclear antigen. These findings together indicate that hUC-MSCs have the capability to differentiate into epithelial-like cells that are normally derived from the endoderm, implicating their potential applications in tissue repair and regeneration of many endoderm-derived internal organs.

## Introduction

Human mesenchymal stem cells (MSCs) are multipotent stem cells found in several adult tissues [1]. These cells are reported to be capable of differentiating into various cell types, particularly the mesoderm-derived tissues including the bone, cartilage, muscle, ligament, tendon, and adipose [2]–[4]. Currently, the most common source of adult MSCs which have a great therapeutic potential is from the bone marrow (BM) because of their capacity of self-renewal and multi-lineage differentiation [5]–[9]. However, there is a great need to identify alternative MSCs sources due to the limited number of BM-MSCs available for autologous uses, the invasive procedures of aspiration of BM, and a significant decrease of frequency and differentiation potential of BM-MSCs as age proceeds [10]. Recent studies have reported an attractive, alternative tissue source of MSCs from human umbilical cord (hUC) [11].

Human UC-MSCs have generated a great deal of interest for their potential use in regenerative medicine and tissue engineering due to their superior advantages compared to the MSCs from the BM. The hUC contains two arteries and one vein, which are surrounded by mucoid connective tissues called Wharton's jelly (WJ) [12]. WJ possesses desirable characteristics such as a large, rapidly available MSCs pool, a non-invasive and painless collection procedure, and ethically non-controversial source of MSCs [13]. In addition, it is believed that the hUC-MSCs are more primitive or less immunogenic than the MSCs derived from other tissue sources, and are endowed with more superior plasticity and a greater expansion capability [14].

Although hUC-MSCs have been shown, as MSCs from the bone marrow, to be able to differentiate into mesodermal tissues such as the bone, cartilage, muscle, ligament, tendon, and adipose, whether they have the capability to differentiate into epithelial cells of endodermal origin such as the prostate epithelial cells is not determined. The prostate is formed through epithelial budding from the urogenital sinus (UGS) derived from the endoderm around days 17–18 of gestation in the mouse [15], [16]. The gland undergoes extensive ductal outgrowth and branching, which continue for several weeks after birth [16]. In humans, budding of the prostatic epithelium is seen at 10 weeks of gestation [17]. The prostate is an important male accessory sex gland found only in mammals that functions to produce a major fraction of seminal fluid, which contains secretory protein prostate specific antigen (PSA).

In the present study, we isolated hUC-MSCs and rat urogenital sinus stromal cells (rUGSSs) and co-transplanted them into renal capsules in vivo. We demonstrated clearly that hUC-MSCs have the capability to differentiate into prostate epithelial-like structures. These structures display similar epithelial lumen, branching patterns as seen for normal prostates, which express prostate-specific markers including PSA. Thus, the hUC-MSCs may have important implications for repair/regeneration of epithelial tissues of endoderm-derived organs.

## Materials and Methods

### Animals

Eighteen-day-pregnant SD rats and male BALB/c nude mice (postnatal day 5 weeks-old) were purchased from Shanghai SLAC Laboratory Animal Co., Ltd. All experiments were approved by the Animal Research Ethics Committee of Renji Hospital, Shanghai Jiao Tong University School of Medicine.

### Antibodies

Antibodies were purchased from the following sources: PE conjugated CD105, FITC conjugated CD29, APC conjugated CD31, PerCP-Cy5.5 conjugated CD45, and PE-Cy7 conjugated CD34 were from eBioscience. p63, AR, CK8, CK5, PSA and vimentin antibodies were from Santa Cruz Biotechnology. Testosterone and collagenase IV were from Sigma and human nuclei antibody was from Millipore.

### Preparation of Dissociated urogenital sinus stromal cells

The rUGSSs isolation procedure was done as previously described [18]. Briefly, E18 embryos from pregnant SD rats were sacrificed and urogenital sinuses were collected. After separation of the UGS from the urogenital sinus epithelium, the cells were digested with 1 mg/ml collagenase IV combined with 0.125% Trypsin for 30 min at 37°C, washed twice and triturated in the culture medium (DMEM supplemented with 10% FBS, 2 mM glutamine, 100 U/ml penicillin and 100 mg/ml streptomycin) and cultured in the same medium in plates. rUGSSs were passaged at confluency by trypsin digestion and cultured in vitro for up to 2 weeks before they were used for tissue recombination with hUC-MSCs prior to transplantation into the renal capsules in the nude mice.

### Isolation and preparation of hUC-MSCs

Fresh hUC were collected from abdominal delivery operation of Renji hospital with the consent of the parents and stored in normal saline. The study protocol was approved by the ethics review board of Renji hospital (protocol #2012-01). We have obtained written informed consent from all study participants. All of the procedures were done in accordance with the Declaration of Helsinki and relevant policies in China. The tissue was disinfected with 70% alcohol for 30 s and cleared extensively with normal saline. The washing was repeated until they were cleaned from blood or blood clots. In order to avoid any contamination of the cultures by the endothelial cells, the vein was flushed with saline prior to the openning at the external surface of the umbilical cord with a sterile scalpel. The cord vessels (arteries and vein) were removed from cord segments, and the exposed WJ tissue was cut into small pieces [19]. After having been minced into 1–2 mm^3^ fragments, WJ tissues were incubated with 1 mg/ml collagenase type IV combined with 0.125% Trypsin for 30 min at 37°C. Following digestion, the mixture was then centrifuged at 400 g for 8 min, washed twice in PBS. WJ cells were placed in plates and cultured in DMEM supplemented with 8% (v/v) fetal bovine serum (FBS), 2 mM L-glutamine, 100 U/ml penicillin, 100 mg/ml streptomycin. The culture plate was placed in an incubator with saturated humidity at about 37°C containing 5% (v/v) CO_2_. Human UC-MSCs were passaged at confluency by trypsin digestion and cultured in vitro for up to 2 weeks.

### Flow cytometry

Once 80% confluence had been reached, adherent cells were analyzed using standard flow cytometry. Compensation adjustments were performed with single color positive controls. Briefly, P0-P8 hUC-MSCs were stained with several antibodies (CD105, CD29, CD45, CD34, CD31) and analyzed on a BD FACSAria II cytometer.

### rUGSSs and hUC-MSCs cultures and immunostaining analyses

rUGSSs and hUC-MSCs were grown on glass coverslips and fixed in 4% paraformaldehyde (for CK8, p63, CK5, AR, PSA, vimentin, human nuclear antigen immunostaining). The cells were then incubated with primary antibody in a humidified chamber overnight at 4°C, processed with secondary antibodies for 1 hr at room temperature in dark. The immunostaining were observed and images were acquired on a Nikon microscope with a digital camera.

### Prostate regeneration assays *in vivo*

The prostate regeneration was performed as previously described [18]. hUC-MSCs (100,000 cells per graft) were mixed with rUGSSs (250,000 cells per graft) in 3 mg/ml collagen type I (20 µl per graft), incubated at 37°C for 1 h to allow collagen gelation, and overlaid with culture medium. After incubation overnight at 37°C, collagen gel was grafted under the renal capsule in 6-8-week-old BALB/c nude mice, along with a subcutaneous slow-release testosterone pellet (12.5 mg per pellet per mouse). Grafts were harvested and analyzed 2 months after implantation. All animal studies were approved by and conducted according to the guidelines of the Renji Hospital Animal Care and Ethics Review Committee.

### Immunohistochemistry

BALB/c nude mouse bearing prostate grafts under the kidney capsule were sacrificed 2 months after surgery. Optimal cutting temperature (OCT) compound-frozen tissues were sectioned at 8 µm, fixed in 4% paraformaldehyde. Sections were stained with hematoxylin/eosin [20]. For immunohistochemistry, sections were incubated with the primary antibody in a humidified chamber overnight at 4°C and secondary antibodies for 1 hr at room temperature in dark. Images were acquired on a Nikon microscope with a digital camera.

## Results

### Isolation and Characterization of hUC-MSCs and rUGSSs

**Figure 1:**
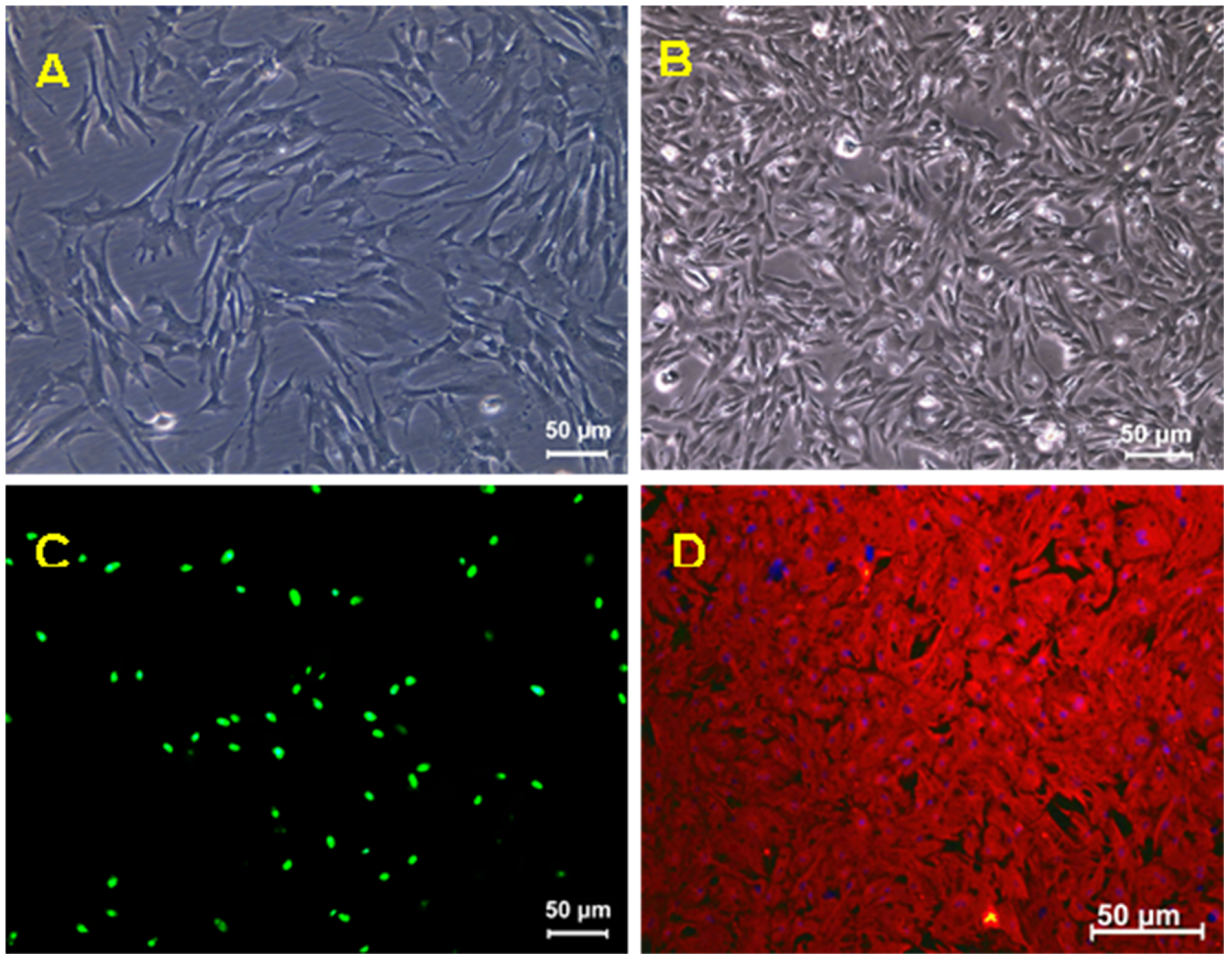
The morphology and characterization of hUC-MSCs and rUGSSs. (A), Seven days after the initial plating, adherent cells derived from hUC displayed a fibroblastic morphology. (B), rUGSSs were small and fibrous in appearance. (C), hUC-MSCs were immunostained by an anti-human nuclei monoclonal antibody (green). (D), rUGSSs were immunostained by vimentin antibody (red). Scale bar: 50 µm.

By enzyme digestion method, cells from human UC fragments were seeded at a density of 1000 cells/cm^2^. As early as 24 h following plating, adherent cells with fibroblastic morphology could be observed and they were hUC-MSCs (Fig. 1A). Rat rUGSSs were prepared from E17-E18 rat embryos as previously described [18]. As compared to the hUV-MSCs, rUGSSs are smaller in size (Fig. 1B). While immunofluorescent staining for human nuclei antibody which recognizes nuclei of all the human cell types labeled all hUC-MSCs (Fig. 1C), all rUGSSs were negative for as expected (data not shown). In addition, the rUGSSs expressed vimentin (Fig. 1D), confirming the stromal cell identity of the rUGSSs.

**Figure 2:**
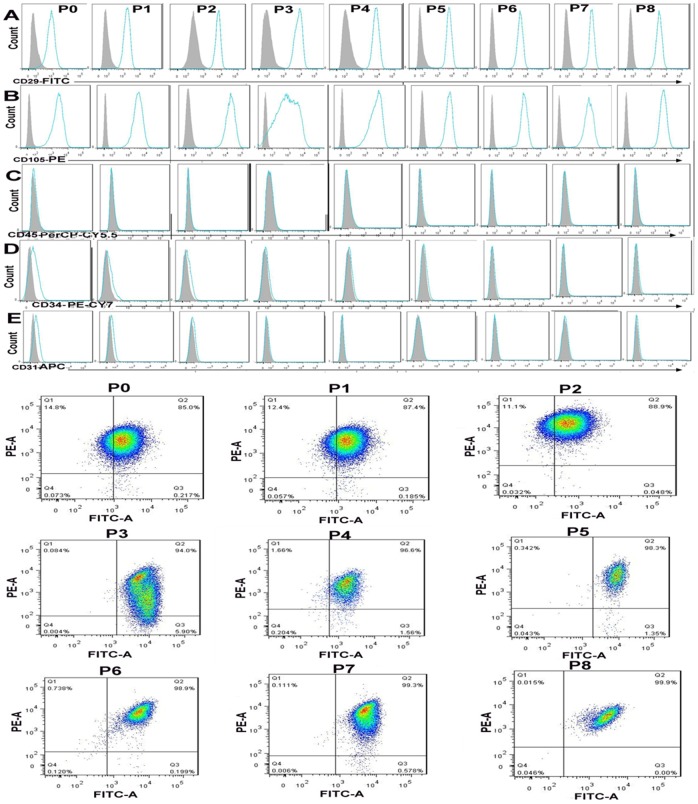
Immunophenotype analysis of P0-P8 hUC-MSCs by FACS. Cells were from P0-P8 hUC-MSCs and stained with CD29, CD105, CD34, CD45, and CD31 antibodies. The upper half of the figure is the flow histogram of single antibody staining. The shaded area shows the profile of the negative control. The lower half of the figure is CD29-FITC and CD105-PE expression of P0-P8 hUC-MSCs. (A) hUC-MSCs exhibit positive surface expression of integrin marker (CD29), (B) MSC marker (CD105), (C-E) but are negative for hematopoietic lineage markers (CD34, CD45) nor the platelet/endothelial cell adhesion molecule (CD31). The CD29+CD105+ hUC-MSC population accounts for 85%∼99.9% of all cells.

To further characterize the hUC-MSCs that we prepared, we analyzed the cells by flow cytometry at P0-P8. As shown in Figure 2, hUC-MSCs exhibited positive surface expression of integrin marker (CD29) (Fig. 2A), MSCs marker (CD105) (Fig. 2B), but were negative for hematopoietic lineage markers (CD45, CD34) (Fig. 2C, D) nor the platelet/endothelial cell adhesion molecule (CD31) (Fig. 2E). The CD29+CD105+ hUC-MSCs population accounted for 85%–99.9% of the total cells.

### Prostate Generation Assays

**Figure 3:**
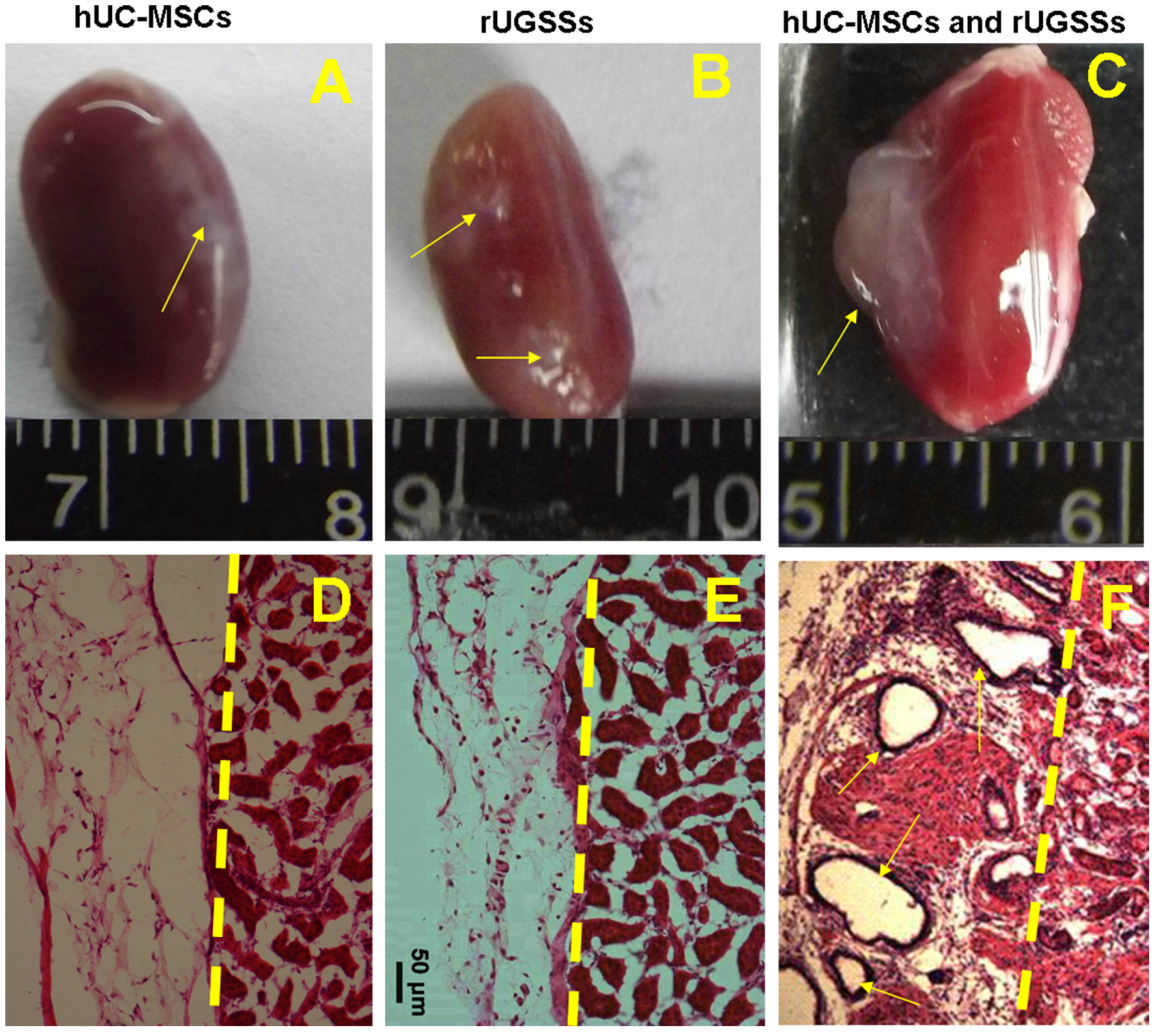
Human UC-MSCs combined with rUGSSs can generate prostate glands. Mice were sacrificed 2 months after co-transplantation surgery, and the kidneys from the cell implanted nude mice were collected. (A) Graft initiated with hUC-MSCs alone and (B) rUGSSs alone were used as negative control, respectively. (C) Graft derived with hUC-MSCs and rUGSSs. (D–F) Histological analyses of the sections of the graft stained for haematoxylin and eosin (H&E). (D) Note that while hUC-MSCs alone and (E) rUGSSs single cell type transplantation fail to regenerate prostate glandular structures. (F) co-transplantation of hUC-MSCs and rUGSSs gives rise to prostate glandular structures. Scale bar 50 µm.

To determine whether they have the capability to differentiate into epithelial cells of endodermal origin such as the prostate epithelial cells, we performed renal capsule cell recombination prostate generation assays. Cells tend to have more robust growth under the kidney capsule, most likely due to the rich blood supply and inductive microenvironment in that area [21], [22]. rUGSSs provide the critical paracrine factors needed for induction of hUC-MSCs growth and development into prostatic epithelial cells. We co-transplanted the hUC-MSCs and rUGSSs into the sub-renal region in nude mice. Grafts were allowed to develop for a period of 2 months before harvest and analysis. We also transplanted solely rUGSSs and solely hUC-MSCs as control experiments. We found that these grafts from sole rUGSSs or hUC-MSCs were small and fibrous in appearance (Fig. 3A, B) and microscopically, they showed a cluster of cells without obvious epithelial glandular structures (Fig. 3D, E). However, in sharp contrast, when the hUC-MSCs were combined with rUGSSs and co-transplanted under the kidney capsule, the grafts formed large translucent epithelial-like glandular structures (Fig. 3C), resembling closely the prostate epithelial acini. These tubular epithelial-like structures in the grafts also displayed lumen filled with fluid (Fig. 3F).

**Figure 4:**
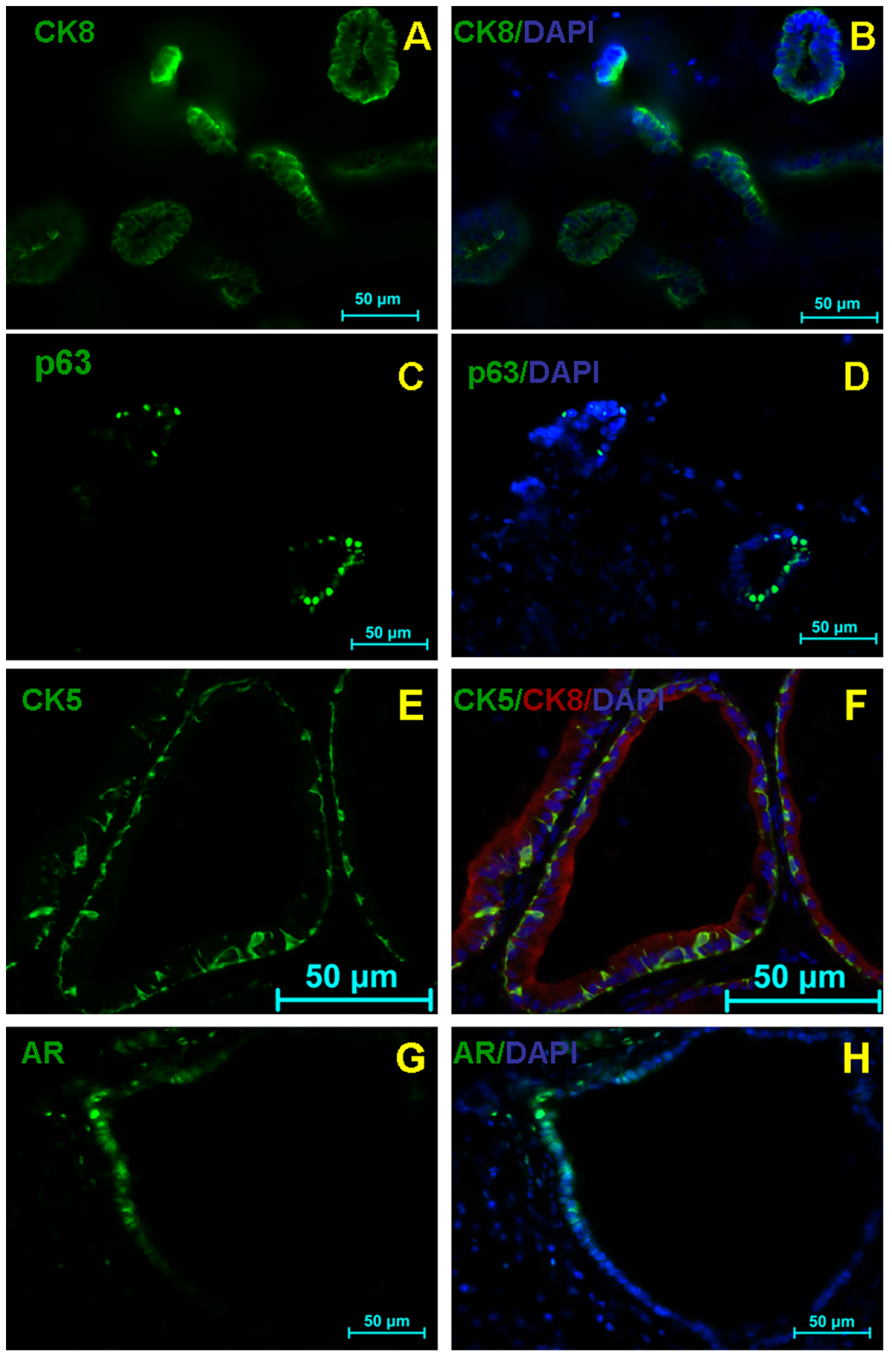
Regenerated prostates resemble the phenotypes of normal prostates. (A, C, E, G) Immunofluorescence analysis of the expression of CK8, p63, CK5 and androgen receptor (AR) in regenerated prostate tissue. (F) shows triple-staining for the basal cell marker CK5 (green), luminal marker CK8 (red) and DAPI counter staining (blue). (B, D, F, H) The tissue sections were counterstained with 4, 6-diamidino-2-phenylindole (DAPI; blue). Scale bar 50 µm.

**Figure 5:**
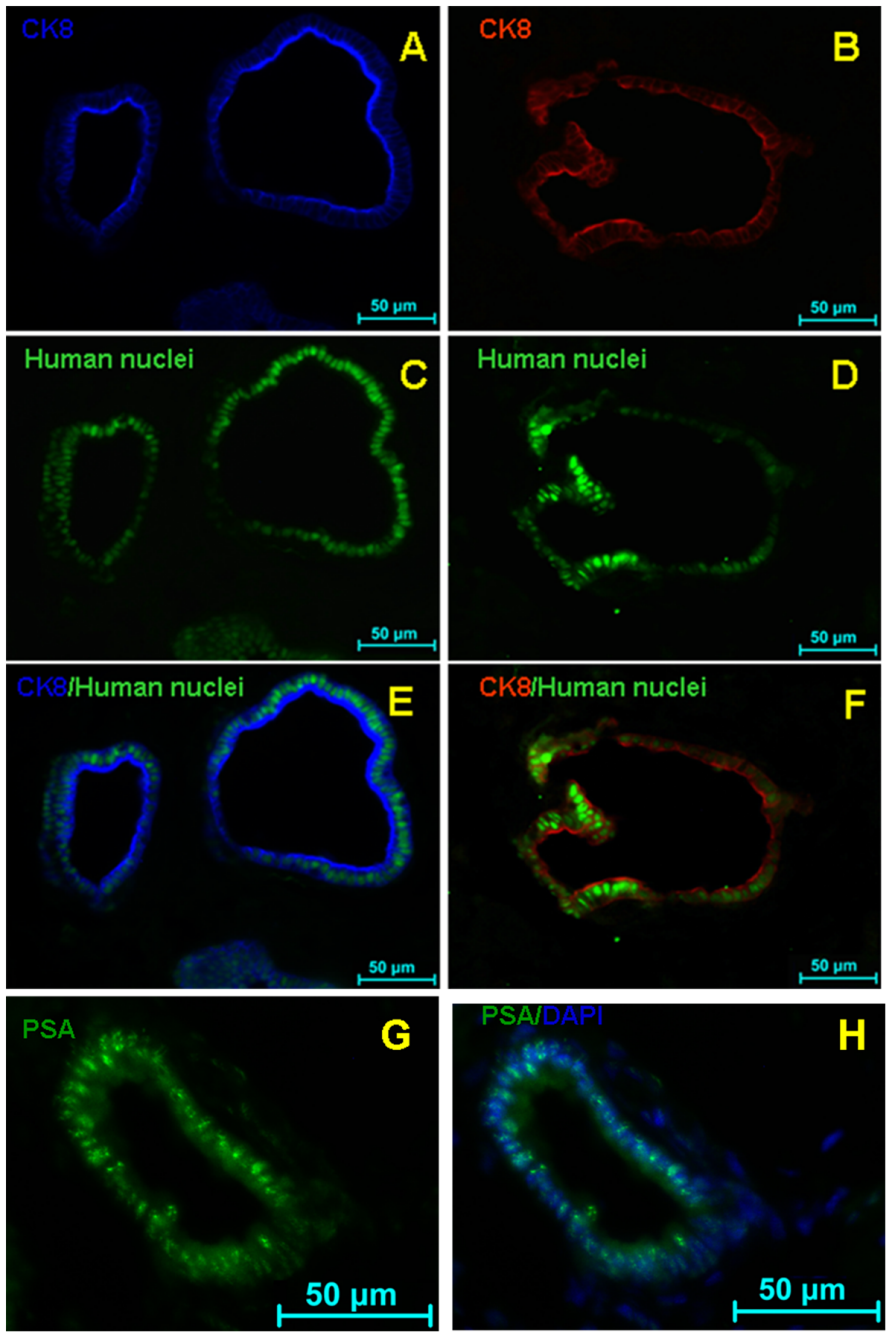
Detection of human cells in the regeneration of prostate. The field of prostate epithelial regeneration can be seen within the graft transplanted with hUC-MSCs and rUGSSs. (A, B) Immunofluorescent staining for CK8 in the new graft. (C, D) Detection of human cells in the new graft (green). (E, F) Note that the human nuclear antigen+ cells (green) in the graft can be co-stained with CK8. (G, H) The grafts express the prostate specific antigen (PSA). Scale bar 50 µm.

Detailed immunohistochemical characterization revealed that the grafts expressed prostate luminal cell marker CK8 (Fig. 4A, B, F and Fig. 5A, B), basal cell marker p63 (Fig. 4C, D) and androgen receptor (Fig. 4G, H). Figure 4E shows single staining for the basal cell marker CK5 (green) and Figure 4F shows triple-staining for the basal cell marker CK5 (green), luminal marker CK8 (red) and DAPI counter staining (blue). In addition, the human origin and functionality of the regenerated grafts were confirmed by expression of anti-human nuclear antigen. As shown in Figure 5, some human nuclear antigen positive cells(Fig. 5C, D) in the graft were double labeled by CK8 antibody (Fig. 5E, F), Furthermore, the epithelial-like cells derived from the grafted hUC-MSCs displayed secretory function as they were specifically labeled by the prostate specific antigen (PSA) antibody (Fig. 5G, H). Of 30 renal capsule grafts with co-transplanted hUC-MSCs and rUGSSs, four showed the formation of prostate epithelial glandular structures. In sharp contrast, in 30 control mice in which sole rUGSSs (15 mice) or sole hUC-MSCs were transplanted (15 mice), none of them displayed any prostate epithelial glandular structures even though some grafted cells survived (Figure 3D, E). Taken together, these findings indicate that the hUC-MSCs had differentiated into prostate epithelial-like cells under these experimental conditions.

## Discussion

Using a cell recombination procedure previously developed by Cunha and Lung [23] or Li et al [18]. We have shown in the present study that a prostate branching structure (Fig. 3F) with epithelial-like tubules composed of basal (p63, CK5; Fig. 4C, E) and luminal (CK8; Fig. 4A, F) cell lineages is formed after co-transplantation of hUC-MSCs together with rUGSSs under renal capsule. The grafts express the prostate specific antigen (PSA) (Fig. 5G) and androgen receptor (AR) (Fig. 4G), indicating generation of a functional prostate. Using a human nuclear antigen antibody, we have verified that the newly generated prostate epithelial-like cells in the grafts were of human origin and not due to contaminating rat epithelial cells from the rUGSSs preparations. We have shown in the present study that hUC-MSCs have the potential to differentiate into prostate epithelial-like cells in vivo. The prostate generation rate from the hUC-MSCs is about 13%. While hUC-MSCs are originated from mesoderm [24], the prostate epithelial cells are derived from the endoderm [25]. Although previous studies have reported that hUC-MSCs can differentiate into mesodermal tissue such as the bone, cartilage, muscle, ligament, tendon, and adipose, to our knowledge, the present work is the first report to clearly demonstrate the capability of hUC-MSCs to differentiate into the endoderm-related prostate epithelium.

Consistent with previous findings, the rUGSSs appear to provide a stem cell niche, which provides necessary signals for the development and generation of prostate structures [23]. During normal embryogenesis, the prostate develops from the urogenital sinus (UGS), a structure derived from bifurcation of the cloaca. The UGS is a midline structure with an endodermally derived epithelial layer (UGE) surrounded by a mesodermally derived stromal cell layer [25]. As with many other tissue, prostate formation is initiated as a consequence of interactions between epithelial and surrounding stromal tissues [16], [20], [26]–[31]. Such rUGSSs appear to provide a stem cell niche that promotes the hUC-MSCs to differentiate into prostate epithelial-like cells. The nature and signaling molecules involved in this process remain to be determined.

Our histological and immunnocytochemical characterization of the grafts provide strong evidence that the glandular structures formed following co-transplantation of hUC-MSCs and rUGSSs are composed of prostatic epithelial-like cells. The generated prostate tissues show the branching epithelial-like morphology and expression pattern of specific marker genes similar to that of normal prostate tissues. The cells in the graft express specific markers for the phenotypically distinct populations of differentiated epithelial cells within the normal prostate. The secretory luminal cells express PSA and cytokeratins 8 [32]–[35] while the basal cells express p63 and CK5 and are located in the basal layer [36]. In addition, the epithelial-like cells also express androgen receptor, an important marker for prostate epithelial cells. Although there is a third, minor population of prostate epithelial cells, called neuroendocrine cells that express synaptophysin, we failed to observe synaptophysin positive cells in the grafts formed, suggesting that either hUC-MSCs do not have to potential to differentiate into this population or the number of this population is too small for us to detect.

It is important to note that not only the cells in the grafts express prostate luminal and basal epithelial cells markers, they also express AR and produce PSA. These findings indicate that the prostate structures formed in the renal capsule are functional. In addition, using an antibody that recognizes specifically the nuclear antigen of human cells, we confirmed that the prostate structures formed are indeed from the hUC-MSCs, rather than from the recipient nude mouse cells. Double immunohistochemical analyses show that human nuclear antigen positive cells in the graft are co-labeled with CK8 antibody, indicating that the CK8 positive luminal cells are derived from hUC-MSCs. Interestingly, we also found that some hUC-MSCs can differentiate into only luminal cell type, suggesting a possibility that some of the hUC-MSCs may follow an unipotent luminal cell lineage while others take a bipotential or multipotential route [37]–[40]. However, the mechanisms controlling the unipotency and bi- or multi-potency of stem cells during prostate development and regeneration require more studies.

Taken together, this study adds to the advancement demonstrating that a functional organ can be generated from a given type of stem cells. Our study clearly demonstrates that stem cells of the mesodermal origin can generate cells/tissue of the endodermal origin. Thus hUC-MSCs might potentially be useful for repair/regeneration of a broader type of tissues.
